# Macrocycle-Assisted Cooperative Fe^3+^ Coordination and Fluorescence Regulation in a Coumarin-Functionalized Calix[4]arene

**DOI:** 10.1021/acsomega.6c02160

**Published:** 2026-04-30

**Authors:** Ömer Güngör, Neşe Taşci, Ayça Şeyma Ünaldı, Mahmut Durmuş

**Affiliations:** † Polymer Science and Technology, Natural Sciences Institutes, 52980 Kocaeli University, Umuttepe Campus, 41001 Kocaeli, Turkey; ‡ Department of Chemistry and Chemical Processing Technologies, Advanced Vocational School of Hereke Asım Kocabıyık, Kocaeli University, 41800 Kocaeli, Turkey; § Department of Chemistry, Faculty of Basic Sciences, 52962 Gebze Technical University, Gebze, 41400 Kocaeli, Turkey

## Abstract

A coumarin-functionalized calix[4]­arene was designed and synthesized as a supramolecular fluorescent platform for the selective recognition of Fe^3+^ ions in aqueous media. The preorganized calix[4]­arene cavity, combined with the coordinating oxygen- and nitrogen-donor sites of the coumarin unit, enables effective host–guest interactions with Fe^3+^, resulting in pronounced fluorescence quenching. Spectroscopic investigations revealed that the quenching process proceeds through a combination of static complex formation and dynamic collisional pathways, supported by Stern–Volmer analysis. Job’s plot analysis indicated a 1:2 binding stoichiometry (Fe^3+^/ligand), highlighting the cooperative role of the calixarene framework in metal-ion coordination. The sensor exhibits a low detection limit of 0.30 μM and a wide linear response range from 33 to 354 μM toward Fe^3+^, while maintaining a high selectivity in the presence of competing metal ions. The applicability of the supramolecular system was further demonstrated through the determination of Fe^3+^ ions in real water samples with satisfactory recovery values. This study illustrates how the integration of a coumarin fluorophore into a calix[4]­arene scaffold provides an effective coordination-driven fluorescence modulation platform, offering insight into calixarene-based metal-ion recognition systems relevant to supramolecular and coordination chemistry.

## Graphical abstract

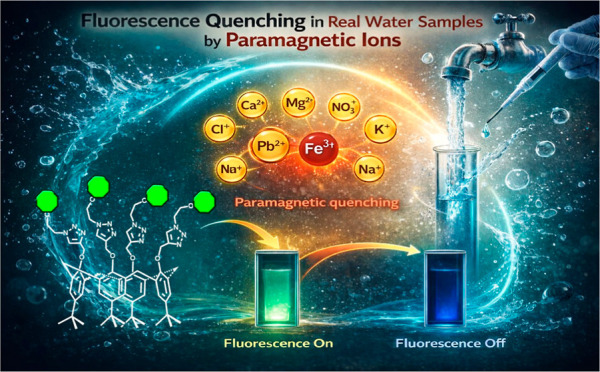

## Introduction

The selective recognition of metal ions using molecularly engineered host systems remains a central theme in supramolecular and coordination chemistry.^1–3^ Among various metal ions, Fe^3+^ occupies a particularly important position owing to its essential biological functions and its strong coordination behavior arising from a high charge density and paramagnetic character. These properties make Fe^3+^ an attractive yet challenging target for molecular recognition, as its coordination-driven electronic effects often lead to a pronounced modulation of photophysical properties in fluorescent systems. Consequently, the rational design of supramolecular platforms capable of translating Fe^3+^ coordination events into reliable optical signals continues to attract significant interest.^4–9^

Iron, which is among the heavy metals, occupies a unique position due to its dual nature as both a vital trace metal and potentially toxic element. Iron plays a fundamental role in numerous physiological and biochemical processes, including oxygen transport, electron and proton transfer, DNA and RNA synthesis, enzymatic catalysis, and cellular respiration. As the second most abundant trace element in the human body, iron is indispensable for almost all living organisms. However, iron is nonbiodegradable and can accumulate naturally in the environment and food chain, posing serious ecological and health risks when present in excessive amounts. Both iron deficiency and iron overload are associated with a wide range of pathological conditions, such as anemia, neurodegenerative disorders (including Alzheimer’s and Parkinson’s diseases), hemochromatosis, cardiovascular diseases, diabetes, liver dysfunction, cancer, and oxidative stress-related cellular damage.^10–22^ In this context, the World Health Organization (WHO) has set the maximum allowable concentration of iron in drinking water at 0.3 mg/L, underscoring the need for precise iron monitoring.^9,23^

The determination of trace levels of Fe^3+^ in real samples has therefore attracted considerable scientific attention. Conventional analytical techniques such as atomic absorption/emission spectroscopy (AAS/AES), inductively coupled plasma mass/atomic emission spectrometry (ICP–MS/ICP-AES), gas chromatography, plasmon resonance Rayleigh scattering (PRRS), and magnetic resonance imaging (MRI) provide high accuracy and reliability.^1,4,24,25^ Nevertheless, these methods are often limited by high operational costs, complex instrumentation, long-term analysis, high energy consumption, and the need for trained personnel.^11,25,26^ As a result, there is a growing demand for alternative sensing approaches that are cost-effective, portable, rapid, and capable of on-site analysis with high sensitivity and selectivity.

Fluorescent chemosensors have emerged as powerful alternatives for Fe^3+^ detection due to their simplicity, fast response, and excellent detection limits. Various fluorescence modulation mechanisms have been employed in Fe^3+^ sensing, including photoinduced electron transfer (PET), ligand-to-metal or metal-to-ligand charge transfer (LMCT/MLCT), internal charge transfer (ICT), chelation-enhanced fluorescence (CHEF), excited-state intramolecular proton transfer (ESIPT), photoinduced proton transfer (PPT), and C=N isomerization.^5^ In particular, fluorescence “turn-off” systems are frequently observed for Fe^3+^ owing to its strong paramagnetic character and high Lewis acidity, which facilitate efficient fluorescence quenching.

In recent years, numerous colorimetric and fluorometric probes based on different fluorophores—including BODIPY,^27^ rhodamine,^12,28,29^ indole,^30^ anthracene,^25^ pyrene,^9,31^ carbazole,^10,32^ phosphazene,^33^ and coumarin^34,35^ derivatives—have been developed for Fe^3+^ recognition. Despite significant progress, many of these systems still suffer from inherent drawbacks, such as poor water solubility, insufficient selectivity against competing metal ions, low sensitivity, slow response times, limited reversibility, and small Stokes shifts. Therefore, the rational design of novel fluorescent sensors that combine high selectivity, sensitivity, structural simplicity, and good photophysical performance for Fe^3+^ detection remains an important challenge.

Calixarenes have attracted considerable attention as versatile supramolecular hosts and have been widely employed in molecular recognition and sensing applications involving azo dyes, pesticides, amino acids, primary amines, and various aromatic compounds.^36–41^ Calix[4]­arenes, in particular, represent highly adaptable supramolecular platforms for sensor design owing to their preorganized cavity, conformational flexibility, and ease of functionalization at both the upper and lower rims.^42–44^ When calix[4]­arene frameworks are coupled with coumarin fluorophores, the resulting hybrid systems benefit from the excellent photophysical properties of coumarin, such as high fluorescence quantum yields, good photostability, and relatively large Stokes shifts. Moreover, the carbonyl oxygen- and heteroatom-containing functional groups (O/N donors) present in the coumarin moiety can act as effective coordination sites for Fe^3+^ ions, which exhibit strong Lewis acidic character. Upon coordination with Fe^3+^, coordination-induced electronic perturbations and paramagnetic effects promote nonradiative deactivation pathways, leading to efficient fluorescence quenching. Consequently, calix[4]­arene–coumarin conjugates enable integrated host–guest recognition and signal transduction within a single molecular architecture, making them promising candidates for selective and sensitive Fe^3+^ detection.^45,46^

In this work, we report the design and synthesis of a coumarin-functionalized calix[4]­arene as a supramolecular fluorescent system for Fe^3+^ recognition. The calix[4]­arene framework provides a preorganized cavity that promotes cooperative metal–ligand interactions, while the coumarin unit serves as both a fluorescent reporter and a coordinating moiety through its oxygen- and nitrogen-donor sites. The interaction between Fe^3+^ ions and the calixarene–coumarin architecture leads to efficient fluorescence quenching, enabling a detailed investigation of binding stoichiometry, quenching behavior, and coordination-driven photophysical modulation. Beyond its analytical response, this study highlights the role of calix[4]­arene-based molecular architectures as versatile platforms for understanding metal-ion recognition and fluorescence regulation within a supramolecular and coordination chemistry context.

Although several coumarin-based fluorescent probes and calixarene-derived sensing systems for Fe^3+^ detection have been reported, most previously described platforms rely on simple 1:1 coordination models or limited linear detection ranges and often lack detailed investigation of cooperative supramolecular binding effects. In particular, reports integrating calix[4]­arene scaffolds with coumarin fluorophores typically focus on signal generation rather than elucidating the contribution of the macrocyclic cavity to binding stoichiometry and coordination-driven fluorescence modulation.

In contrast, the present study introduces a preorganized calix[4]­arene–coumarin architecture that enables cooperative 1:2 (Fe^3+^/ligand) complex formation, as confirmed by Job’s plot analysis, and demonstrates a dual static–dynamic fluorescence quenching mechanism. The wide linear working range and low detection limit achieved in this system highlight the analytical relevance of the supramolecular design. Beyond serving as a fluorescent sensor, this work provides insight into how macrocyclic preorganization and multidentate coordination sites can synergistically regulate photophysical behavior, offering a structurally defined platform for coordination-driven fluorescence modulation.

## Experimental Section

### Materials and Instruments

The calix-coumarin synthesis procedure is given in Scheme 1. The used materials, equipment, and photophysical and photochemical parameters are given in the Supporting Information.

**Scheme 1:**
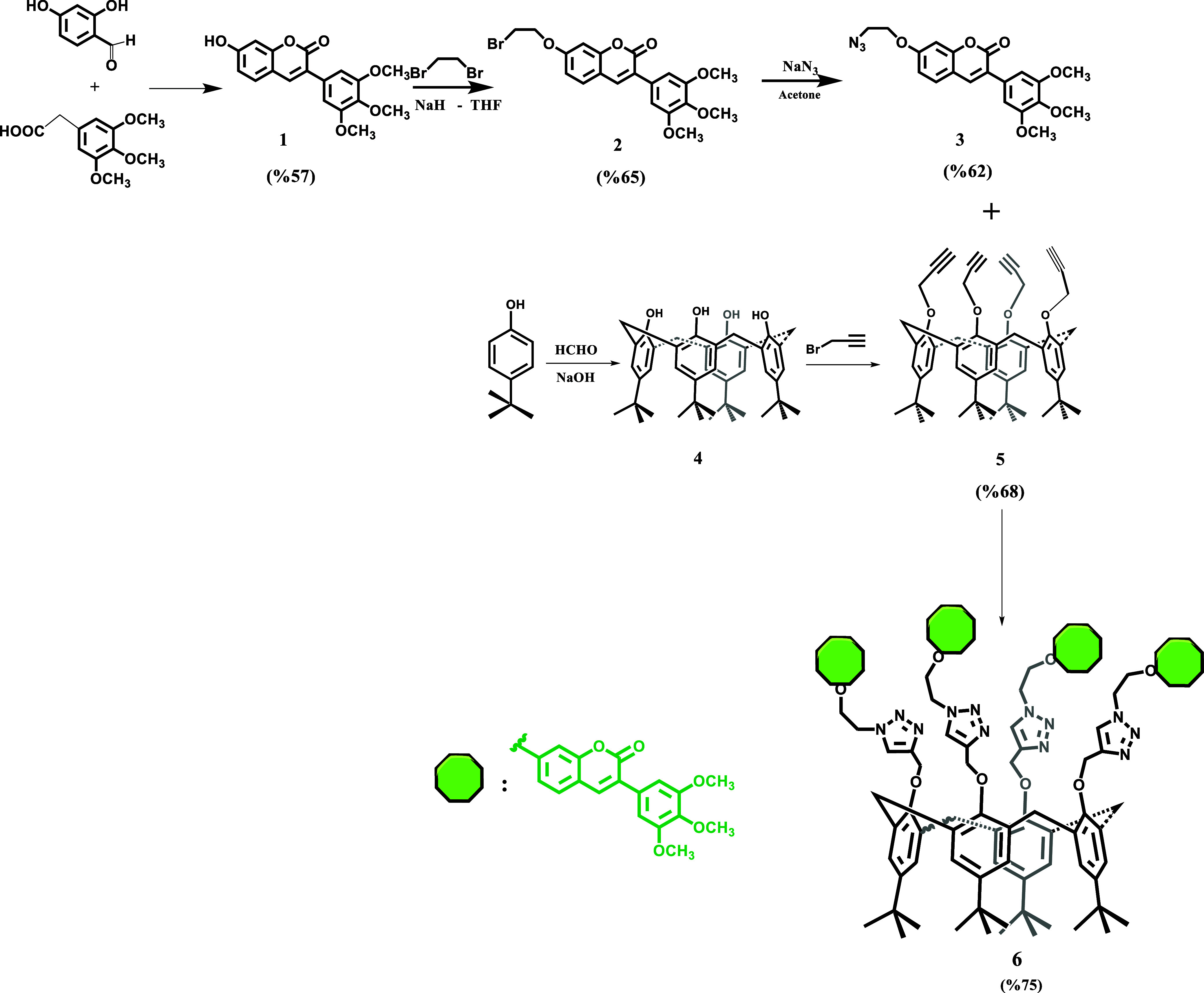
Synthesis of Calix-Coumarin Derivative

### Experimental Section

The *p*-*tert*-butylcalix­[4]­arene (**4**), 7-Hydroxy-3-(3′,4′,5′-trimethoxyphenyl) coumarin (**1**) and 5,11,17,23-tetra-*t*-butyl-25,26-bis­(O-propargyl) calix­[4]­arene (**5**) were synthesized according to the procedures given in the literature.^47–49^

### The Synthesis of 7-(2-Bromoetoksi)-3-(3′,4′,5′-trimethoxyphenyl)­coumarin (**2**)

Compound **1** (3.05 mmol, 1.00 g), 1,2-dibromoethane (60.92 mmol, 5.25 mL), and K_2_CO_3_ (3.05 mmol, 0.42 g) were dissolved in 60 mL of dry acetone and refluxed for 48 h. The reaction was monitored by Thin Layer Chromatography (TLC). Subsequently, the mixture was extracted three times with 20 mL of CH_2_Cl_2_. The filtrate was dried over Na_2_SO_4_, and the solvent was evaporated to dryness. The solid residue was purified by silica gel column chromatography, using CH_2_Cl_2_/hexane (1:2) as the eluent. *R*_*f*_: 0.60. Compound 2 was obtained as a pale yellow solid; yield: 45%; mp 132–133 °C. FT-IR (ν_max_/cm^–1^): 2940 (aromatic, C–H), 2834 (aliph. C–H), 1707 (C=O). ^1^H NMR (500 MHz; DMSO-*d*_6_): δH, ppm: 3,69 (s, 3H, -OCH3), 3,80 (s, 6H, -OCH3), 3,91 (t, 2H, CH_2_–Br), 4,25 (t, 2H, OCH_2_), 6,11 (dd, j:8,5 and 2,3 Hz, 1H, Ar–H), 7,01 (d, 1H), 7,05 (s, 2H, lactone-H), 7,68 (d, j:8, 5 Hz, 1H, Ar–H), 8,23 (s, 1H, Ar–H). MS (ES^+^), (*m*/*z*): calcd for: C_20_H_19_O_6_Br, 435.26; found, 435.020 [M]^+^, 458.340 [M + Na]^+^.

### The Synthesis of 7-(2-Azidoetoksi)-3-(3′,4′,5′-trimethoxyphenyl)­coumarin (**3**)

Compound **2** (2.30 mmol, 1.00 g) was dissolved in 15 mL of DMSO and then sodium azide (4.59 mmol, 0.30 g) was added to the reaction mixture. The reaction mixture was stirred at 65 °C under reflux for 12 h. The reaction was monitored by Thin Layer Chromatography (TLC). Upon completion of the reaction, the mixture was extracted three times with 20 mL of CH_2_Cl_2_, and the organic phase was dried over Na_2_SO_4_. The solvent was evaporated by using a rotary evaporator. Compound **3** was obtained as a yellow solid; yield: 85%; mp 136–137 °C. FT-IR (ν_max_/cm^–1^): 3100 (aromatic C–H), 2948–2840 (aliph. C–H), 2126 (C–N3 stretching), 1724 (C=O). ^1^H NMR (500 MHz; DMSO-*d*_6_): δH, ppm: 3,71 (s, 3H, –OCH_3_), 3,72 (t, 2H, CH_2_–N_3_), 3,83 (s, 6H, –OCH_3_), 4,32 (t, 2H, OCH_2_), 7,01 (d, 1H, Ar–H), 7,05 (s, 2H, lactone-H), 7,10 (dd, j:8,5 and 2,3 Hz, 1H, Ar–H), 7,70 (d, j:8,5 Hz, 1H, Ar–H), 8,25 (s, 1H, Ar–H). MS (ES^+^), (*m*/*z*): calcd for: C_20_H_19_O_6_N_3_, 397,38; found, 397.050 [M]^+^, 418.574 [M + Na]^+^.

### The Synthesis of Compound **6** (Calix-Coumarin)

Compound **3** (1.26 mmol, 0.50 g) was dissolved in 20 mL of dry toluene. Then, compound **5** (0.32 mmol, 0.25 g), CuI (0.32 mmol, 0.12 g), and triethylamine (0.64 mmol, 0.19 g) dissolved in 20 mL of DMF were added to the reaction medium. The reaction mixture was refluxed for 24 h. At the end of the reaction, it was extracted 3 times in the water-CH_2_Cl_2_ system after cooling to room temperature. The filtrate was dried in anhydrous MgSO_4_. The solvent was evaporated by the evaporator and crude product was purified in the ethyl acetate/hexane (1:2) system on a silica gel column. FT-IR (ν_max_/cm^–1^): 3010–2926 (aromatic C–H), 2900 (aliph. C–H), 1721 (C=O), 1121,8 (Ar–O–Ar). ^1^H NMR (500 MHz; DMSO-*d*_6_): δH, ppm: 1.16 (s, 36H, *t*-butyl), 3.15 (d, 4H, *J* = 12.6 Hz, Ar–CH_2_–Ar), 3.74 (t, 8H, *J* = 10.1 Hz, CH_2_–N) 3.70 (s, 12H, –OCH_3_), 3.83 (s, 24H, –OCH_3_), 3.91 (s, 8H), 4.35 (t, 8H, OCH_2_), 4.62 (d, 4H, *J* = 12.8 Hz, Ar–CH_2_–Ar), 7.00 (s, 8H, Ar–H), 7.09 (s, 8H, lactone-H), 7.04 (d, *J* = 11.2 Hz, 4H, Ar–H), 7.14 (s, 4H, Ar–H), 7.72 (s, 4H, triazole), 7.73 (d, *J* = 8.5 Hz, 4H, Ar–H), 8.27 (s, 4H, Ar–H). MS (ES+), (*m*/*z*): Cal. for: C_136_H_140_N_12_O_28_, 2390,62; Found: 2390,67 [M]^+^, 2454,70 [M + Cu]^+^.

## Results and Discussion

### Synthesis and Characterization of the Coumarin-Modified Calix[4]­arene (**6**)

The coumarin-modified calix[4]­arene sensor was synthesized through a stepwise functionalization strategy designed to preserve the calixarene framework while introducing the fluorescent signaling unit in a controlled manner. The synthetic route afforded the target compound in a satisfactory yield and purity, demonstrating the robustness and reproducibility of the proposed methodology. The molecular structure of the synthesized sensor was unambiguously confirmed by a combination of spectroscopic techniques, including FT-IR, ^1^HNMR, and mass spectrometry.

In the FT-IR spectrum, the successful incorporation of the coumarin moiety was evidenced by the appearance of a characteristic carbonyl stretching band in 1721 cm^–1^, attributable to the lactone C=O group of the coumarin unit. Additionally, the aromatic C=C stretching vibrations observed around 1600 cm^–1^, together with the disappearance or attenuation of reactive precursor bands, further supported the formation of the desired coumarin-functionalized calix[4]­arene structure.

The ^1^H NMR spectrum provided clear insight into the structural integrity of the calix[4]­arene scaffold and its substitution pattern. The presence of well-resolved aromatic proton signals corresponding to both the calixarene phenyl rings and the coumarin moiety confirmed successful conjugation. The characteristic methylene bridge protons of the calix[4]­arene appeared as distinct signals, indicating that the macrocycle predominantly adopts a cone conformation, which is favorable for host–guest interactions. ^1^H NMR spectroscopy is a versatile and reliable tool for determining the conformational behavior of calix[4]­arene derivatives. The ^1^H NMR spectrum of calix-coumarin 6 exhibited a typical AB pattern corresponding to the methylene bridge protons (ArCH_2_Ar) of the calixarene framework, appearing at 3.15 and 4.62 ppm with coupling constants of *J* = 12.6 and 12.8 Hz, respectively. This characteristic AB system confirms that the compound adopts a cone conformation. The *tert*-butyl groups of the calixarene unit showed a singlet at 1.16 ppm corresponding to thirty-six aliphatic protons. The aromatic protons associated with the coumarin moiety and triazole rings appeared as multiplet signals in the region of 8.27–7.04 ppm. In addition, the aromatic protons of the calixarene framework were observed as characteristic signals centered at around 7.00 ppm. Furthermore, the methoxy group protons of the coumarin units were detected as singlet peaks at 3.70 and 3.83 ppm, which is consistent with the proposed structure of calix-coumarin. Overall, the ^1^H NMR data strongly support the successful synthesis and cone conformation of the calix-coumarin structure.

Mass spectrometric analysis further confirmed the formation of the target compound by displaying a molecular ion peak corresponding to the expected molecular weight of the coumarin-modified calix[4]­arene. Taken together, these characterization results unequivocally verify the successful synthesis of the designed fluorescent sensor and provide a solid structural basis for subsequent investigations of its photophysical behavior and Fe^3+^ sensing performance. Spectra of the compounds and further interpretations are provided in the Supporting Information section.

### Spectroscopic Properties of Calix-Coumarin

The effect of solvent on the absorption and fluorescence properties of Calix-coumarin derivative was investigated in different solvents (toluene, dichloromethane (DCM), dimethyl sulfoxide (DMSO), chloroform, ethanol, and methanol). As shown in Figure 1a,b, when different solvents were used, the absorption band did not change much but the fluorescence intensity was changed depend on the variety of the solvents. The results suggested that ethanol is the most suitable solvent for further photophysicochemical investigation of the Calix-coumarin compared to other solvent. Therefore, ethanol was used as the solvent for follow-up studies.

**Figure 1:**
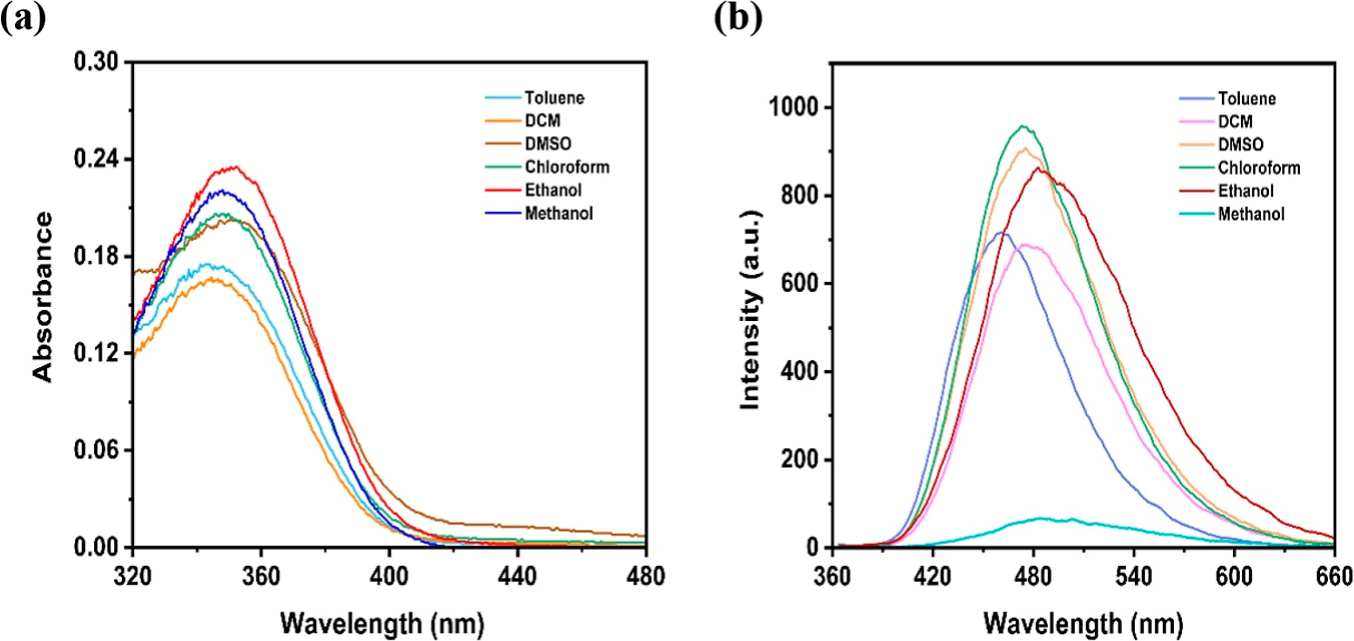
(a) UV–vis electronic absorption spectra (1 × 10^–5^ M). (b) Fluorescence emission spectra (3 × 10^–6^ M) of the Calix-coumarin derivative in different solvents.

Figure 2 illustrates the absorption, emission, and excitation spectra of the Calix-coumarin derivative. The maximum absorption, excitation and emission wavelengths for the Calix-coumarin derivative were observed at 345, 350, and 489 nm, respectively. Thus, Stokes shift was found to be 144 nm for this compound. Fluorescence quantum yield (Φ_F_) measurements for the Calix-coumarin derivative were also done and the value was calculated as 0.103 (Quinine sulfate standard in 0.1 N H_2_SO_4_ with a Φ_F_ value of 0.54 was used as a reference).^50^ Upon the addition of Fe^3+^, the Φ_F_ of the Calix-coumarin–Fe^3+^ complex decreased to 0.034, indicating efficient fluorescence quenching. Additionally, the molar extinction coefficient (ε) was found to be 5 × 10^5^ M^–1^ cm^–1^.

**Figure 2:**
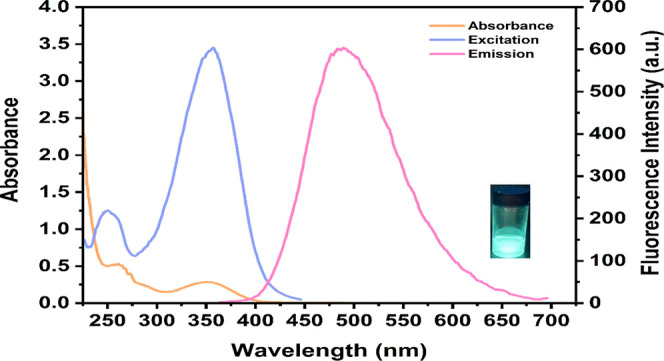
UV–vis electronic absorption, excitation (λ_ex_: 350 nm) and emission (λ_em_: 489 nm) spectra of the Calix-coumarin derivative. The inset figure shows image of the Calix-coumarin derivative in ethanol under a UV lamp.

### Fluorescence Procedure Optimization of the Calix-Coumarin Derivative

The effect of various parameters on the fluorescence procedure of the Calix-coumarin derivative for iron determination such as the primary concentration of the Calix-coumarin derivative, sensitivity, and selectivity of the sensor in the presence of the competitive ions, and the time before detection were perused.

### Selectivity

The selectivity of the fluorescence sensor, particularly in the analysis of real samples, constitutes the most crucial performance parameter. Fluorescence spectroscopy was employed to investigate the selectivity of the Calix-coumarin derivative. All spectral measurements were carried out using a micropipette in a 10 mL spectroscopic quartz cuvette at 25 °C. Stock solutions of nitrate salts of metal ions and the Calix-coumarin derivative were prepared in ultrapure water and EtOH, respectively. In order to identify the selectivity of the developed fluorescence sensor toward Fe^3+^, the impact of interfering species including different metal cations (Na^+^, Al^3+^, Co^2+^, Zn^2+^, etc.) and anions (CH_3_COO^–^, NO_3_^–^, S_2_O_3_^2–^, HPO_4_^2–^, etc.) on the fluorescence intensity of the Calix-coumarin derivative was measured. All ions were measured at the same concentration and under the same condition. Figure 3 shows that the addition of other interfering species (354 μM in water) has a negligible effect on the emission intensity of the calix-coumarin derivative (3 μM in EtOH). Only the addition of Fe^3+^ quenched the fluorescence intensity of the Calix-coumarin derivative. From the diversity of the fluorescence signal of this compound between iron-containing metal and iron-free metal blends can be understood that several competitive species do not affect the Calix-coumarin derivative’s fluorescence signal. The results confirmed that the Calix-coumarin derivative could be a highly selective fluorescence turn-off sensor for the detection of Fe^3+^.

**Figure 3:**
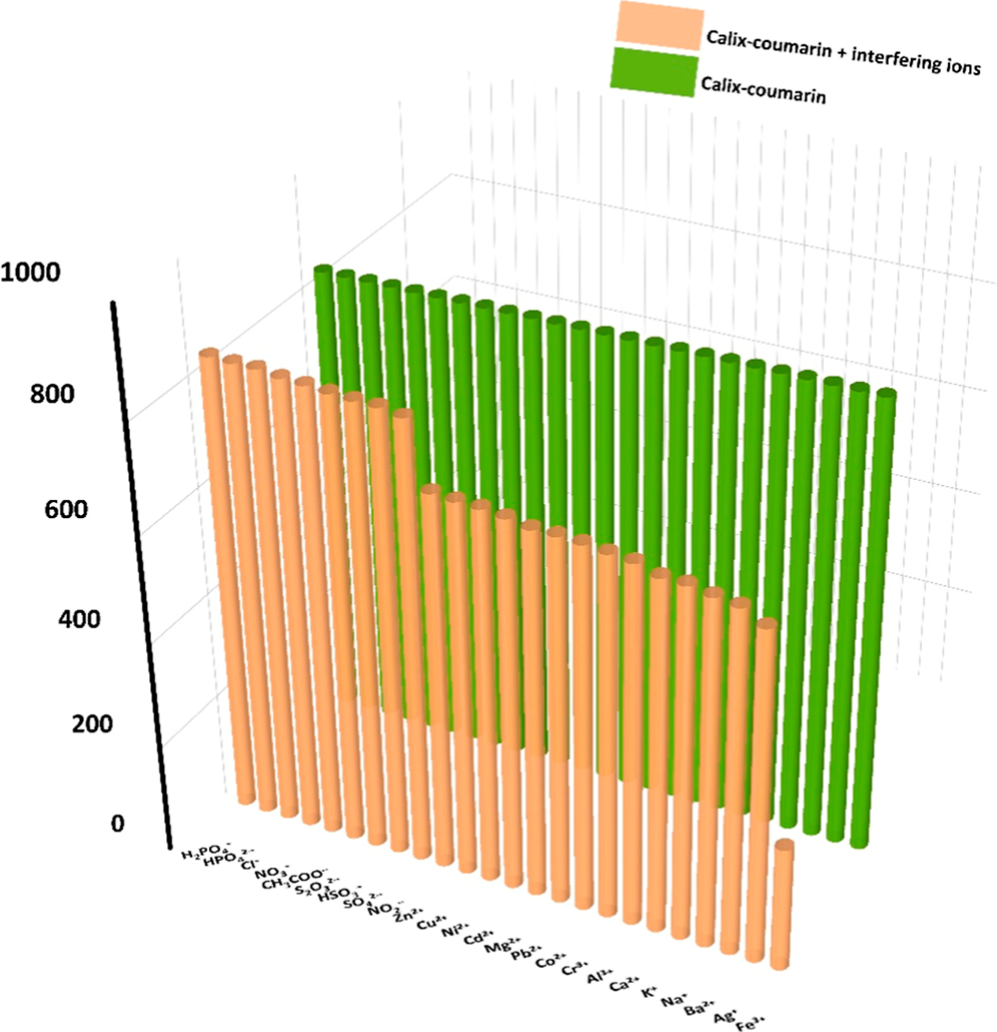
Bar diagram showing the fluorescence responses of the Calix-coumarin derivative upon the addition of various interfering ions (Green bar: the Calix-coumarin derivative and orange bar: Calix-coumarin + interfering ions).

### Influence of the Time before Measurement

One of the parameters affecting the complexation of Fe^3+^ with the Calix-coumarin derivative is the time before measurement. Therefore, the iron determination process was carried out while the time before detection was varied in the range of 0–600 s. The maximum fluorescence intensity, under optimal conditions for all other parameters (3 μM Calix-coumarin in EtOH), was reached at 10 s, after which the fluorescence intensity remained unaffected (Figure 4). Considering the stability of the formed complexes, a waiting time of 10 s before the measurement was adopted.

**Figure 4:**
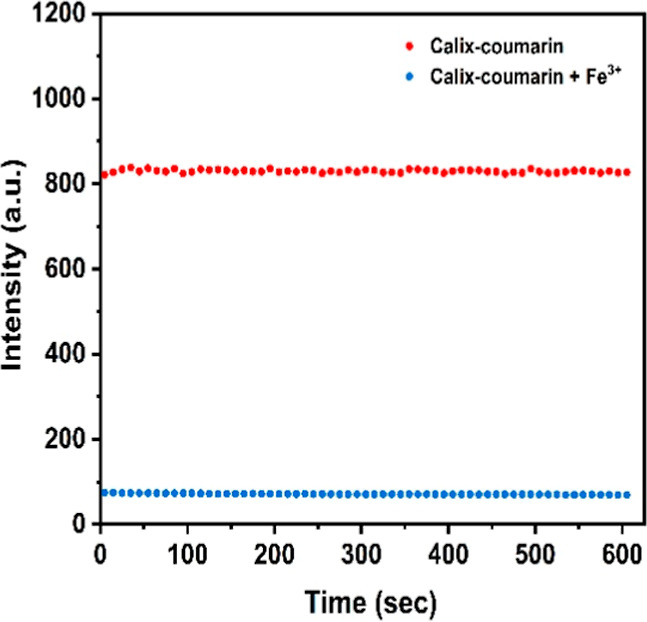
Measurement time on relative fluorescence response of Calix-coumarin + Fe^3+^ in EtOH (λ_ex_ = 350 nm, 3 μM Calix-coumarin and 354 μM Fe^3+^, and slit width = 5 nm).

### Interaction and Fluorescent Quenching Mechanisms of the Calix-Coumarin–Fe^3+^ Complex

The stoichiometry of the Fe^3+^–Calix-coumarin complex was investigated using the method of continuous variation (Job’s plot) in ethanol. The increasing Fe^3+^ mole fraction in the presence of 3 μM Calix-coumarin was utilized to acquire Job’s plot (Figure 5a). As shown by the Job’s plot, the maximum fluorescence change occurs at an Fe^3+^ mole fraction of 0.33. The Job’s plot indicates a 1:2 (Fe^3+^: Calix-coumarin) binding stoichiometry.

**Figure 5:**
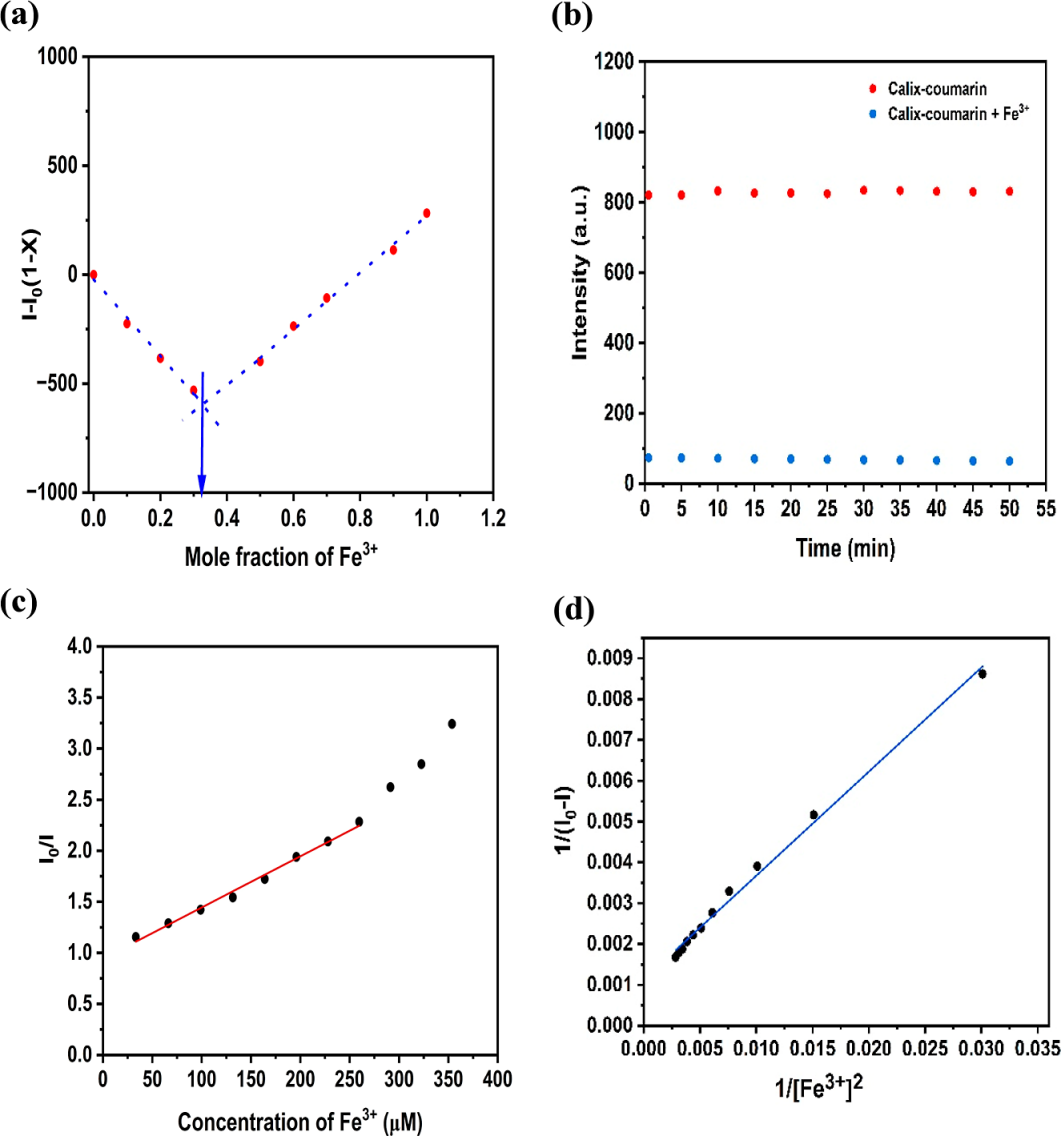
(a) The Job’s plot for determining the stoichiometric ratio between the Calix-coumarin derivative and Fe^3+^, (b) photostability of the Calix-coumarin derivative and Calix-coumarin + Fe^3+^ in EtOH, (c) the Stern–Volmer plot of the Calix-coumarin derivative with the addition of various concentrations of Fe^3+^ (33–354 μM) (λ_ex_ = 350 nm and slit width = 5 nm), and (d) Benesi–Hildebrand (BH) plot for the 1:2 complexation of the Calix-coumarin derivative with Fe^3+^ in EtOH, used to calculate the association constant (*K*_a_).

The 1:2 complexation stoichiometry of the Calix-coumarin derivative with Fe^3+^ was further confirmed by the Benesi–Hildebrand (BH) method. A BH plot was constructed by plotting 1/(*I*_0_ – *I*) versus 1/[Fe^3+^]^2^ for the 1:2 binding model, Excellent linearity (*R* ^2^ = 0.9944) was observed, consistent with Job’s plot analysis (Figure 5d). From the slope and intercept of the BH plot, the association constant (*K*_a_) was calculated as 4.43 × 10^3^ M^–1^ supporting strong host–guest interaction. These results validate the proposed 1:2 binding mode of the Calix-coumarin–Fe^3+^ system.

The macrocyclic calix[4]­arene framework plays a significant role in the fluorescence sensing performance of calix-coumarin 6. Although acyclic coumarin derivatives are known to detect Fe^3+^ ions via fluorescence quenching, these systems generally rely on single-site coordination and often exhibit moderate selectivity. In contrast, calix[4]­arene-based systems provide preorganized cavities, multiple coordination sites, and cooperative binding interactions, which enhance metal-ion recognition and sensing efficiency. The observed 1:2 binding stoichiometry between Fe^3+^ and calix-coumarin 6 further supports the cooperative binding behavior enabled by the macrocyclic scaffold. Therefore, the enhanced fluorescence quenching efficiency and selectivity observed in this study can be attributed to the macrocyclic calix[4]­arene architecture, which promotes stronger and more selective coordination with Fe^3+^ ions.^34,51,52^

To obtain highly accurate and precise results, the photostability of the sensor and complex is a crucial factor. For this purpose, fluorescence emission of the Calix-coumarin derivative was recorded for 50 min in the absence and presence of Fe^3+^ ions. As shown in Figure 5b, the fluorescence of the Calix-coumarin derivative and Calix-coumarin + Fe^3+^ is stable during this 50 min. These results indicated that this sensor and its complex have good photostability.

Fluorescence quenching can generally proceed through two distinct mechanisms, namely, static and dynamic quenching. Static quenching arises from the formation of a nonemissive complex between the fluorescent receptor and the analyte (quencher) in the ground state. In contrast, dynamic quenching occurs as a result of collisional interactions between the quencher and fluorophore molecules after the fluorophore has been promoted to the excited state.^53^ An important factor governing fluorescence intensity is the concentration of the quencher, as its variation provides valuable insight into the underlying quenching pathway. The Stern–Volmer equation (eq 1) is used to investigate the quenching mechanism:

1 $$I_{0}/I=K_{sv}\left[Q\right]+1$$

where *Q*, *I*, *I*_0_, and *K*_sv_, demonstrate the concentration of quencher (in molar), the ultimate fluorescence intensity of the Calix-coumarin derivative in the attendance of the quencher, the primary fluorescence emission of fluorophore (Calix-coumarin), and the constant of Stern–Volmer, respectively. In the case of static quenching, a linear relationship between *I*_0_/*I* and the quencher concentration (*Q*) is obtained with an intercept of unity on the *y*-axis. However, when both static and dynamic quenching processes contribute simultaneously, an upward (positive) deviation from linearity is observed as the quencher concentration increases.^48^ The Stern–Volmer plot corresponding to the interaction between the Calix-coumarin derivative and Fe^3+^ is presented in Figure 5c. The results illustrate a linear fluorescence response with an increasing Fe^3+^ concentration up to 260 μM, beyond which a positive deviation becomes evident for the Calix-coumarin–Fe^3+^ system. Based on these observations, it can be concluded that the fluorescence quenching of the Calix-coumarin derivative involves a combination of static and dynamic quenching mechanisms.

In addition to the mechanistic insights discussed above, the high selectivity, fluorescence quantum yield change, and rapid response of the calix-coumarin derivative upon Fe^3+^ binding were further investigated. The high selectivity of the calix-coumarin derivative toward Fe^3+^ arises from the combined effect of the preorganized calix[4]­arene cavity and the oxygen- and nitrogen-donor sites of the coumarin unit. This architecture allows strong host–guest coordination specifically with Fe^3+^ ions, while other metal ions are unable to interact efficiently due to differences in charge, size, or coordination preferences.

The fluorescence quantum yield (Φ_F_) of the calix-coumarin derivative in ethanol was measured as 0.104. Upon addition of Fe^3+^, the fluorescence was efficiently quenched (Φ_F_ = 0.034), indicating a strong interaction. The response was also very rapid, with maximum quenching observed within 10 s, demonstrating that the sensor was both highly sensitive and fast-responding. These characteristics support the practical applicability of the sensor for Fe^3+^ detection in real water samples.

### Analytical Figures of the Calix-Coumarin Derivative

To determine the linear operating range of the sensor, fluorescence titration experiments were carried out under controlled conditions using a 10 μM Calix-coumarin derivative with incremental Fe^3+^ additions (0–354 μM) in a 3 mL quartz cuvette at 25 °C. Each sample was equilibrated for 10 s before measuring fluorescence emission at 475 nm (λ_ex_ = 350 nm, slit width = 5 nm). As shown in Figure 6a, a linear decrease in fluorescence intensity at 475 nm was observed as the Fe^3+^ concentration increased. Calibration curves of Calix-coumarin + Fe^3+^ have been shown in Figure 6b. As can be seen, the linear range of the sensor was 33–354 μM for Fe^3+^ (*y* = 1.4632*x* + 103.23, where *y*: (*I*_0_ – I), *x*: [Fe^3+^], *R* ^2^ = 0.9932). Also, the sensor’s limit of detection (LOD) and limit of quantification (LOQ) were calculated from the calibration curve using the formulas LOD = 3σ/*S* and LOQ = 10σ/*S*, respectively, by taking three times and ten times the standard deviation of the blank signal intensity. Here, σ is the standard deviation of the Calix-coumarin derivative, and S is the slope of the calibration plot. The LOD and LOQ were found to be 0.30 and 1.00 μM, respectively. United States Environmental Protection Agency (EPA) has reported 0.30 ppm (5.40 μM) in drinking water as the tolerance concentration of iron ions and the achieved LOD and LOQ contents via the introduced sensor are lower than 5.40 μM.^54^

**Figure 6:**
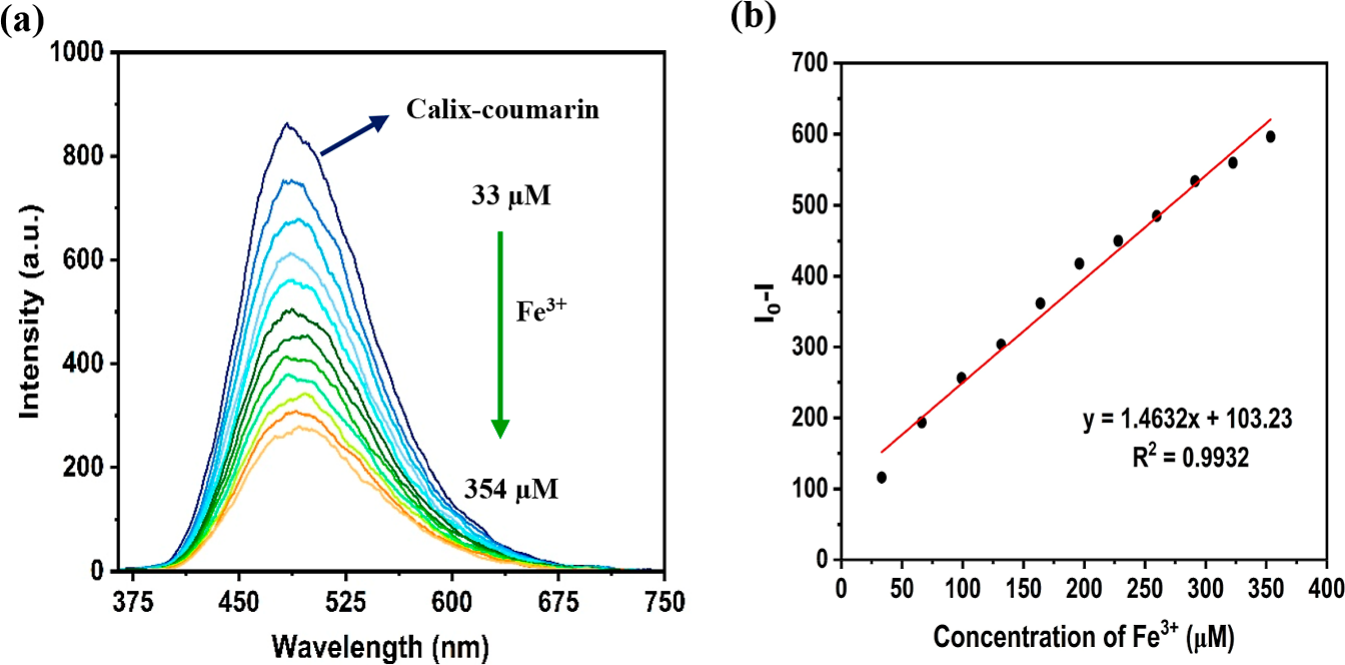
Linear fluorescence response of the calix-coumarin derivative (3 μM) toward increasing concentrations of Fe^3+^ ions in EtOH: (a) fluorescence emission spectra and (b) corresponding calibration curve. Measurements were performed at an excitation wavelength of 350 nm with a slit width of 5 nm.

### Effect of pH on the Sensing Performance

The influence of pH on the fluorescence response of the calix-coumarin system was investigated over a broad pH range (2.0–9.0; Figure 7). This study evaluated the change in fluorescence intensity after adding 150 μM Fe^3+^ ions, prepared at different pH values (2.0–9.0), to 3 μM calix-coumarin. As shown in the figure, the fluorescence intensity decreases upon addition of Fe^3+^, reaching a minimum at around pH ≈ 7.0, indicating maximum quenching efficiency. At lower pH values, the reduced quenching can be attributed to protonation of the oxygen- and nitrogen-donor sites of the coumarin moiety, which diminishes coordination with Fe^3+^. In contrast, at higher pH values, the decrease in available Fe^3+^ due to hydrolysis and formation of insoluble iron hydroxide species results in weaker quenching. These results indicate that the optimal sensing performance is achieved under near-neutral conditions (pH ≈ 7.0), consistent with the experimental conditions used and supporting the applicability of the sensor in real water matrices.

**Figure 7:**
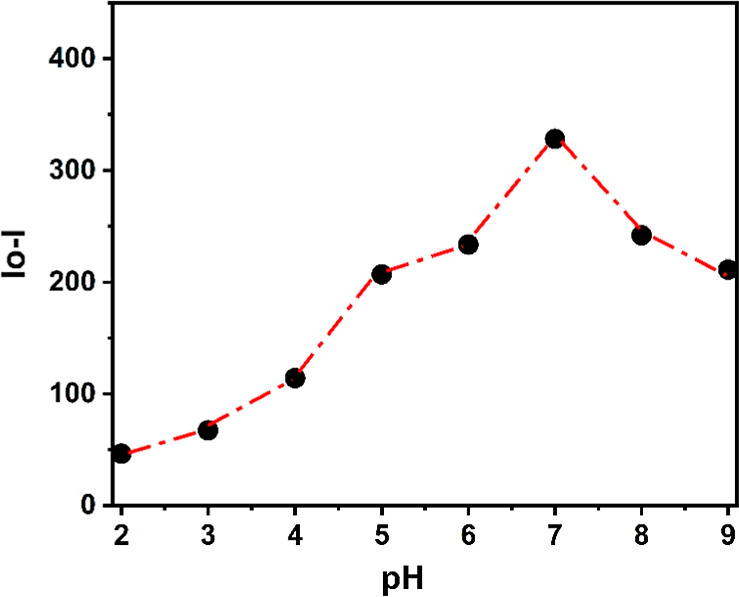
Effect of pH on the fluorescence quenching of the calix-coumarin derivative in the presence of Fe^3+^ ions.

### Precision of the Sensor

Intraday and interday studies were conducted to assess the repeatability of the proposed method. In the intraday study, Fe^3+^ was measured at three different concentrations (61.74, 215.1, and 337.2 μM) with three repetitions within a single day, while in the interday study, each concentration was analyzed three times each day for 3 days. The results are listed in Table 1. Relative standard deviation (RSD) values ranging from 0.64% to 1.11% and 0.37% to 2.37% were obtained for the intraday and interday studies, respectively. The RSD values were within the expectable range, supporting that the method has satisfactory precision.

**Table 1:** Precision Values of the Developed Sensor for Detection of Fe^3+^

	intraday (*n* = 3)			interday (*n* = 3)		
added (μM)	61.74	215.1	337.2	61.74	215.1	337.2
found (μM)	63.1	214.9	337.51	58.4	213.7	335.62
	62.7	217.2	339.74	61.09	213.26	337.19
	61.7	214.7	335.45	60.61	212.15	334.02
mean	62.5	215.59	337.57	60.04	213.03	335.61
SD	0.69	1.41	2.15	1.42	0.79	1.59
RSD (%)	1.11	0.65	0.64	2.37	0.37	0.47

### Applicability of the Sensor to Real Samples

To investigate the real-world application of the Calix-coumarin sensor, the determination of Fe^3+^ ions was evaluated by analyzing water samples using fluorescence emission spectroscopy. For this purpose, spike and recovery tests with Fe^3+^ ions at concentrations of 0, 61.74, 215.05, and 294.34 μM were performed in tap water samples using fluorescent sensors. The results, summarized in Table 2, show recovery values of tap water samples in the range of 98.13 to 117.82% with the RSDs from 0.12% to 0.57%. In the case of well water samples, the recoveries were 91.46–95.02% with RSDs ranging from 0.30–0.98%. The spike and recovery analysis results showed that analytical recoveries were obtained by using the proposed fluorescent sensor, suggesting that the sensor could be a strong candidate for the determination of Fe^3+^ ions in water matrices.

**Table 2:** Spike and Recovery Tests for Fe^3+^ Ions Using the Purposed Sensor in Real Water Samples

sample	spiked (μM)	found (μM)	recovery (%)	RSD (%)
tap water	0	ND^a^	-	-
	61.74	72.75	117.82	0.57
	215.05	220.61	102.58	0.12
	294.34	288.83	98.13	0.24
well water	0	ND	-	-
	61.74	57.75	93.54	0.98
	215.05	204.34	95.02	0.30
	294.34	269.20	91.46	0.80

a Not detected.

A comparison between the “turn-off” fluorescence methods developed in this study and previously reported fluorescence-based approaches for Fe^3+^ ion detection is summarized in Table 3. The results demonstrate that the proposed method offers lower limits of detection (LODs), a wider linear working range, and higher sensitivity and selectivity, while being less affected by interfering species, in comparison with conventional fluorescence technique. Although certain fluorescence methods reported in the literature can achieve lower LOD values, they are often limited by significant interference effects and a restricted linear working range. Overall, the methods presented in this work provide a more reliable and well-balanced analytical performance for Fe^3+^ determination.

**Table 3:** Comparison of the Other Reported and Presented Spectrofluorimetric Determination Methods for Fe^3+^ Ions

material	LOD (μM)	Linear range (μM)	interference	application	ref
Tb-MOF	0.936	0.330–33.00	–	water samples	^26^
Rhodamine B-derivative	Not reported	0–60.00	Al^3+^	Cell imaging	^55^
Naphthalimide-derivative	0.57	0–9.00	Hg^2+^	Not reported	^56^
Hydroxyquinoline	3.00	5.01–10.03	–	bovine liver	^57^
Pyrazole bearing imidazole	1.730	2.000–9.000	Al^3+^	water samples	^58^
Rhodamine-thioxoquinazolinone	4.11	0–75.00	Ag^+^,Hg^2+^, Cu^2+^	Cell imaging	^59^
Rhodamine-derivative	100	Not reported	Cu^2+^, Cr^3+^	Not reported	^60^
Anthracene excimer-based probe	Not reported	Not reported	Cr^3+^	Cell imaging	^61^
Cellulose-based fluorescent paper	Not reported	Not reported	–	naked-eye detection in water	^62^
Green fluorescent carbon quantum dots (CQDs)	Not reported	Not reported	Fe^3+^	Biological systems	^63^
Coumarin-modified Calix-arene	0.30	33–354	–	water samples	This study

As summarized in Table 3, a variety of fluorescent probes have been reported for Fe^3+^ detection, including rhodamine, naphthalimide, hydroxyquinoline, pyrazole, and anthracene derivatives. Many of these systems suffer from limitations, such as narrow linear working ranges, insufficiently reported detection limits, or application-specific restrictions, and some are primarily demonstrated for biological imaging rather than quantitative analysis in aqueous media. Calixarene-based sensors offer structural tunability and preorganized binding cavities, but reported systems often show moderate analytical performance or require complex architectures. In contrast, the coumarin-modified calix[4]­arene sensor developed in this study demonstrates a well-balanced analytical profile, combining a low detection limit (LOD = 0.30 μM) with a wide linear working range (33–354 μM), rapid response (maximum quenching within 10 s), and high selectivity toward Fe^3+^ ions. Moreover, the successful application to real water samples confirms its practical usability. These results clearly highlight the novelty, originality, and excellence of the present sensor compared to previously reported Fe^3+^ chemosensors, providing a reliable and efficient platform for Fe^3+^ determination in aqueous environments.

Calixarene-based fluorescent sensors constitute an important class of supramolecular probes, owing to their preorganized binding cavities and structural tunability. Nevertheless, many reported calixarene-derived systems exhibit moderate analytical performance or limited linear ranges and in some cases rely on complex molecular architectures or auxiliary signal modulation strategies, thereby restricting their applicability in routine analytical measurements, particularly in real water samples.

In contrast, the coumarin-modified calix[4]­arene sensor developed in the present study demonstrates a well-balanced analytical profile, combining a low detection limit (LOD = 0.30 μM) with a wide linear working range (33–354 μM) and reliable fluorescence quenching behavior toward Fe^3+^ ions. The sensing process is based on a simple and reproducible fluorescence “turn-off” mechanism, enabling direct quantitative analysis without the need for multiemission signal processing or advanced data treatment. Importantly, the successful application of the proposed sensor to real water samples further highlights its practical analytical utility. Overall, compared to previously reported fluorescent probes, the present calixarene-based system provides a robust and analytically efficient platform for reliable Fe^3+^ determination in aqueous environments.

## Conclusion

In this paper, a coumarin-modified calix[4]­arene-based fluorescent sensor was successfully designed, synthesized, and evaluated for the selective detection of Fe^3+^ ions. The sensor exhibited an efficient fluorescence quenching response toward Fe^3+^, originating from strong host–guest interactions within the calixarene cavity and paramagnetic quenching effects. Detailed analytical investigations revealed a low detection limit of 0.30 μM along with a wide linear working range of 33–354 μM, highlighting the sensor’s capability for reliable quantitative analysis.

In conclusion, the coumarin-functionalized calix[4]­arene reported in this study demonstrates how a preorganized macrocyclic scaffold can be effectively combined with a fluorescent coordinating unit to achieve coordination-driven modulation of photophysical behavior. The observed fluorescence quenching upon Fe^3+^ binding originates from cooperative metal–ligand interactions within the calixarene framework coupled with the paramagnetic nature of the metal ion. Beyond its analytical response, this system provides insight into the role of calix[4]­arene-based architectures as adaptable supramolecular platforms for metal-ion recognition and fluorescence regulation. The modular nature of the present design also offers opportunities for structural modification toward the development of related coordination-responsive systems, underscoring the broader relevance of this approach within supramolecular and coordination chemistry. Importantly, the present system does not merely function as a fluorescence “turn-off” probe but exemplifies how macrocyclic preorganization and cooperative coordination can be strategically combined to regulate excited-state behavior. The observed 1:2 binding stoichiometry and dual quenching pathway underline the active structural role of the calix[4]­arene framework rather than serving as a passive scaffold. This study therefore contributes to a broader understanding of coordination-driven fluorescence modulation in supramolecular systems and offers a rational design principle for future calixarene-based sensing architectures.

## Supplementary Material



## Data Availability

The data supporting the findings of this study are available within the article and its Supporting Information.

## Notes

FT-IR spectra of synthesized compounds; ^1^H NMR spectra of compounds; mass spectra of synthesized compounds; UV–vis absorption spectra; fluorescence emission spectra and titration data; Job’s plot analysis; Stern–Volmer plots; photostability studies; calibration curve; and analytical performance data (PDF)
