# Supplementary material for: Macrocycle-Assisted Cooperative Fe3+ Coordination and Fluorescence Regulation in a Coumarin-Functionalized Calix[4]arene
Source: ACS Omega. 2026 Apr 30;11(18):27493–503. doi: 10.1021/acsomega.6c02160 (PMC13177233; doi:10.1021/acsomega.6c02160)
Supplement: Supplementary file 1 [file ao6c02160_si_001.pdf]

SUPPORTING INFORMATION

Macrocycle-Assisted Cooperative Fe^{3+} Coordination and Fluorescence Regulation in a Coumarin-Functionalized Calix[4]arene

Ömer Güngör[a,b*], Neşe Taşci[c], Ayça Şeyma Ünaldı[c] and Mahmut Durmuş[c*]

[a]Kocaeli University, Polymer Science and Technology, Natural Sciences Institutes, Umuttepe Campus, 41001, Kocaeli, Turkey

[b]Kocaeli University, Department of Chemistry and Chemical Processing Technologies, Advanced Vocational School of Hereke Asım Kocabıyık, 41800, Kocaeli, Turkey

[c]Gebze Technical University, Department of Chemistry, Faculty of Basic Sciences, Gebze, 41400, Kocaeli, Turkey

***Authors for correspondence**.

Assoc. Prof. Dr. Ömer Güngör, Kocaeli University, Department of Chemistry and Chemical Processing Technologies, Advanced Vocational School of Hereke Asım Kocabıyık, 41800, Kocaeli, Turkey

E-mail: omer.gungor@kocaeli.edu.tr

Prof. Dr. Mahmut Durmuş, Gebze Technical University, Department of Chemistry, Faculty of Basic Sciences, Gebze, 41400, Kocaeli, Turkey

E-mail: durmus@gtu.edu.tr

Materials and instruments

Deionised water passed from Millipore milli Q Plus water purification system was used for the preparation of aqueous solutions. Analytical TLC was performed on precoated silica gel plates (SiO_2, Merck PF_{254}), while silica gel 60 (Merck, particle size 0.040–0.063 mm, 230–240 mesh) was used for preparative column chromatography. The analytical grade organic solvents and other reagents were commercially provided by Merck or from Sigma-Aldrich and were used without any purification. 2-Azidoethanol was purchased from PubChem. UV-Vis spectrophotometer (1800, Shimadzu, Japan) and Spectrofluorometer (Eclipse, Varian, USA) were utilized to obtain UV–vis electronic absorption and steady-state fluorescence measurements, respectively. Slit widths were selected 5 nm and pathlength was 1 cm for all fluorescence experiments. Fourier transform infrared spectroscopy (Perkin Elmer Spectrum 100 ATR-FTIR spectrometer). NMR spectra (1H NMR, 500 MHz) were recorded on a Bruker Fourier-transform spectrometer at 298 K. Chemical shifts (δ, ppm) were referenced to TMS for ^{1}H nuclei measurements.

Characterization of Compound 2

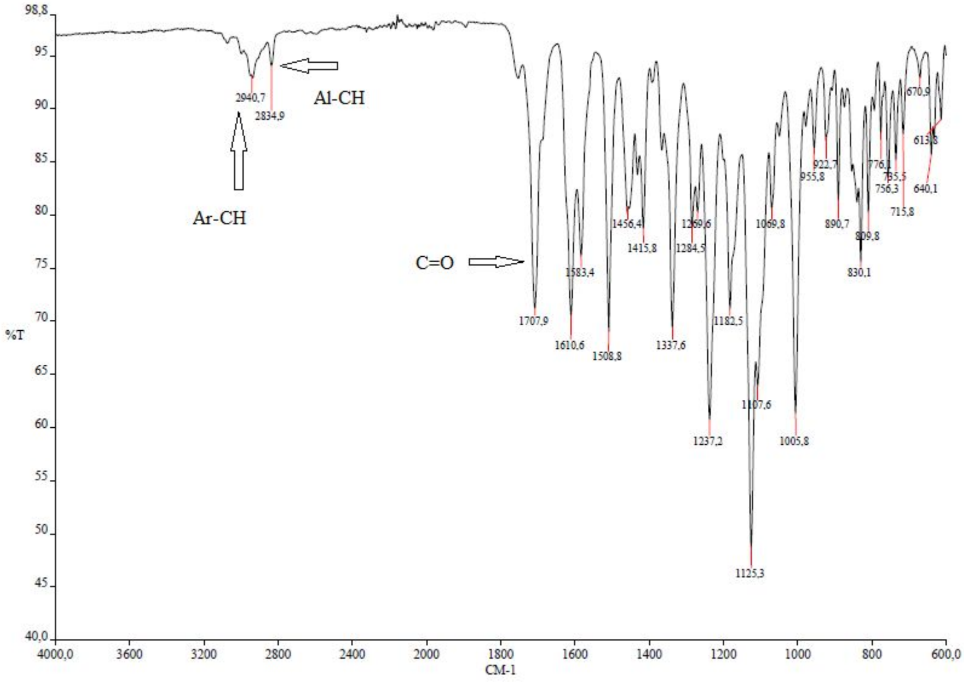

Figure S1. FT-IR Spectrum of Compound **2**.

In the FT-IR spectrum, the disappearance of the OH peak at 3206 cm^{-1} corresponding to coumarin (**1**), the presence of an aromatic -CH peak at 2940 cm^{-1}, an aliphatic -CH peak at 2834 cm^{-1}, a carbonyl (C=O) peak at 1707 cm^{-1}, aromatic C=C peaks in the range of 1610-1456 cm^{-1}, and an ether (Ar-O-Ar) peak in the range of 1237-1125 cm^{-1} confirm the structure of the compound **2**.

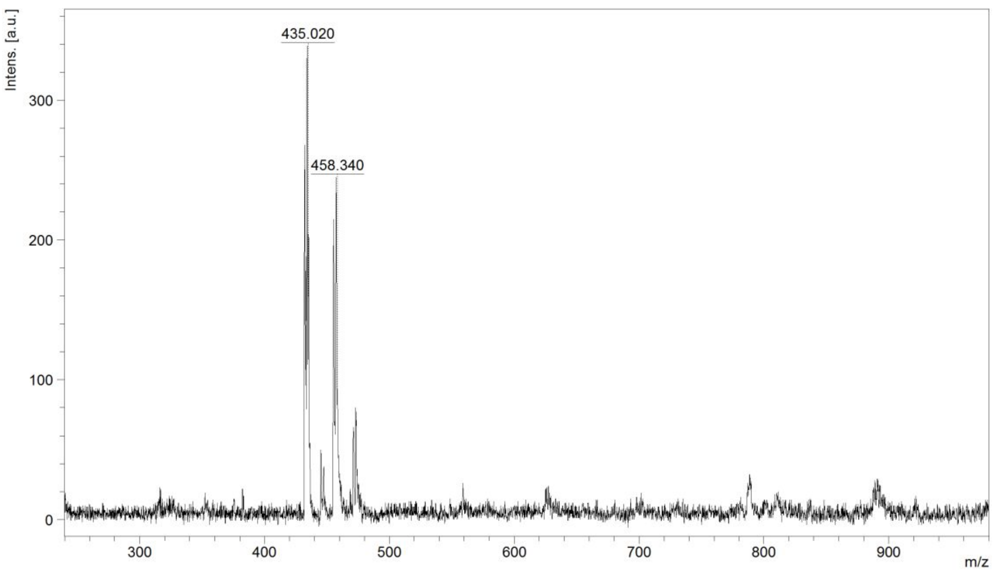

Figure S2. Mass spectrum of the compound **2**.

The mass spectrum of the compound is provided in Figure S2. Upon analysis of the spectrum, the molecular ion peak $[M]^+$ at 435.020 m/z and the $[M+Na]^+$ peak at 458.340 m/z for this compound, which has the molecular formula $C_{20}H_{19}O_6Br$ and a calculated molecular weight of 435.26 g/mol, support the correctness of the expected structure of the compound **2**.

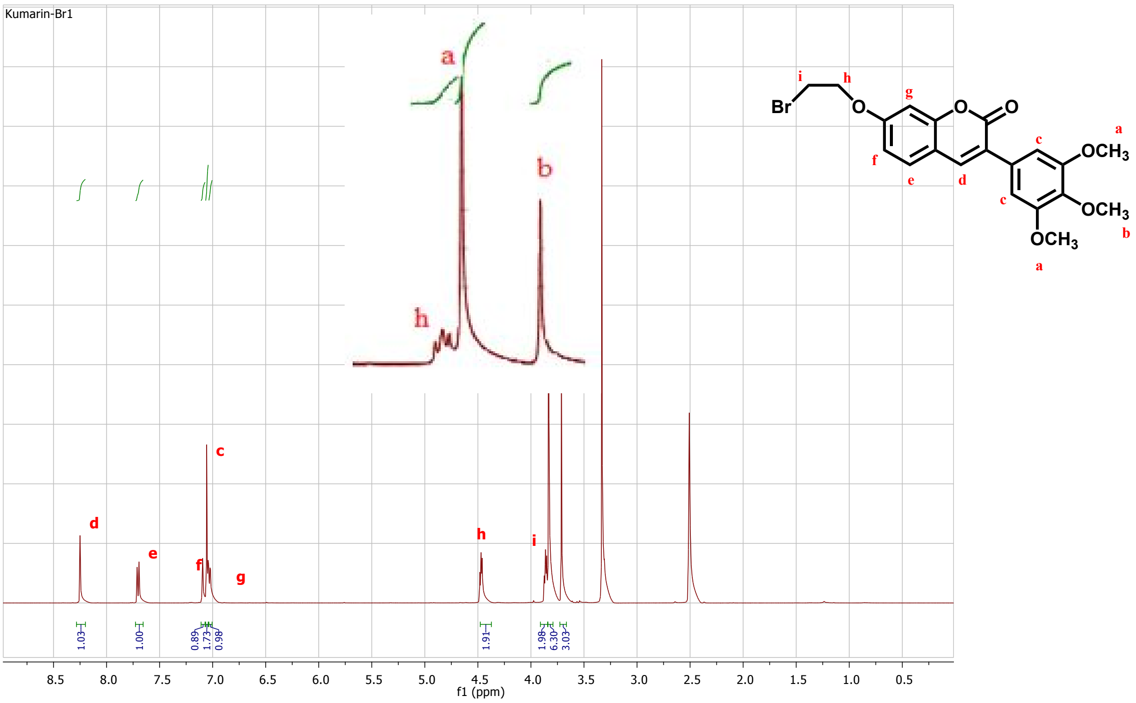

Figure S3. ^{1}H-NMR spectrum of the compound **2.**

In the ^{1}H-NMR spectrum of compound **2** recorded in DMSO-d_6, the aromatic protons were observed in the range of 7.68-7.01 ppm. The He proton appeared as a doublet at 7.68 ppm. The Hf proton, which exhibited ortho coupling with the He proton, split into a doublet, and further split into a doublet of doublets due to meta coupling with the Hg proton, was observed at 6.11 ppm. The Hg proton appeared as a doublet at 7.01 ppm. The Hc protons were observed as a singlet at 7.05 ppm. The Ha and Hb protons of the methoxy groups attached to the meta and para positions of the phenyl ring at the 3-position of the coumarin structure were observed as singlets at 3.80 and 3.69 ppm, respectively. In the ^{1}H-NMR spectrum of the coumarin compound, the vinylic Hd proton was observed as a singlet at 8.23 ppm, downfield. The aliphatic CH_2 protons appeared as triplets at 4.25 and 3.91 ppm, splitting into each other. All observed chemical shift values (δ), coupling constants (J), and integration values for this compound confirm the correctness of the structure.

Characterizations of Compound 3

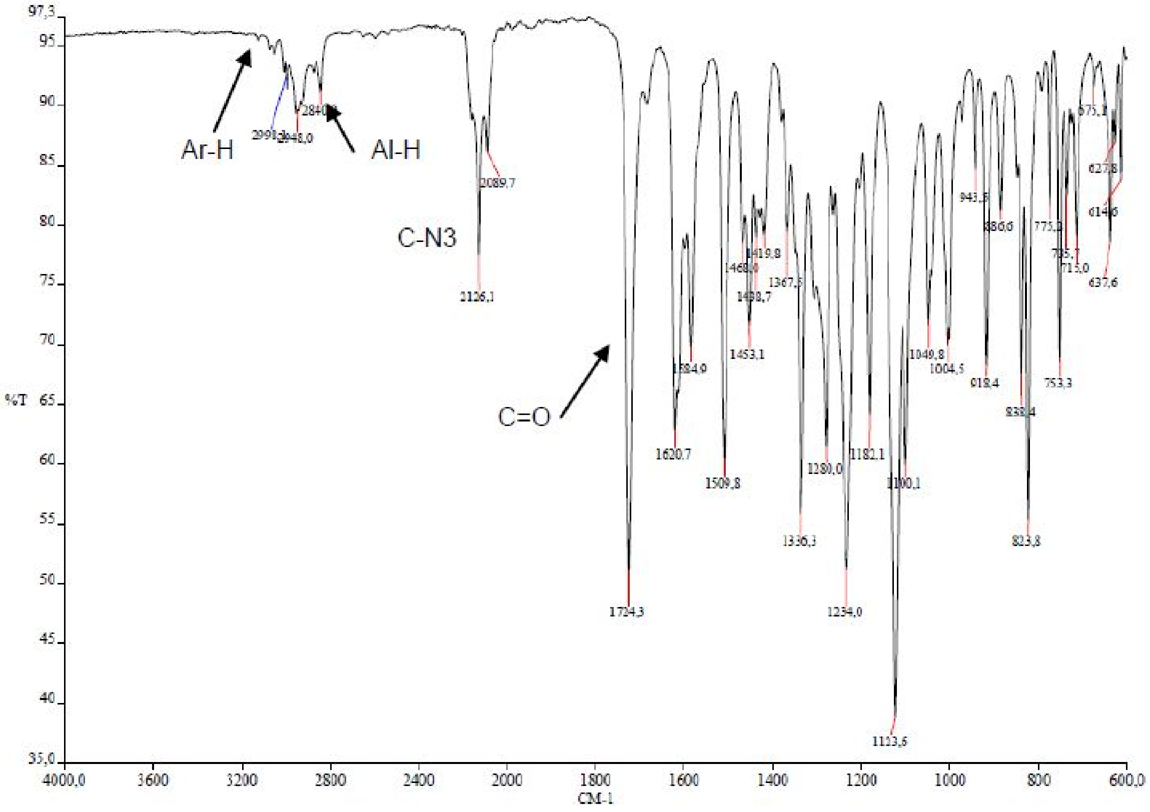

Figure S4. FT-IR spectrum of the compound **3**.

In the FT-IR spectrum of the compound **3**, an aromatic -CH peak was observed at around 3100 cm^{-1}, aliphatic -CH peaks were observed in the range of 2948-2840 cm^{-1}, an azide peak was observed at 2126 cm^{-1}, a carbonyl (C=O) peak was observed at 1724 cm^{-1}, aromatic C=C peaks were observed in the range of 1620-1453 cm^{-1}, and an ether (Ar-O-Ar) peak was observed in the range of 1234-1123 cm^{-1}. These observations confirm the structure of the compound **3**.

.

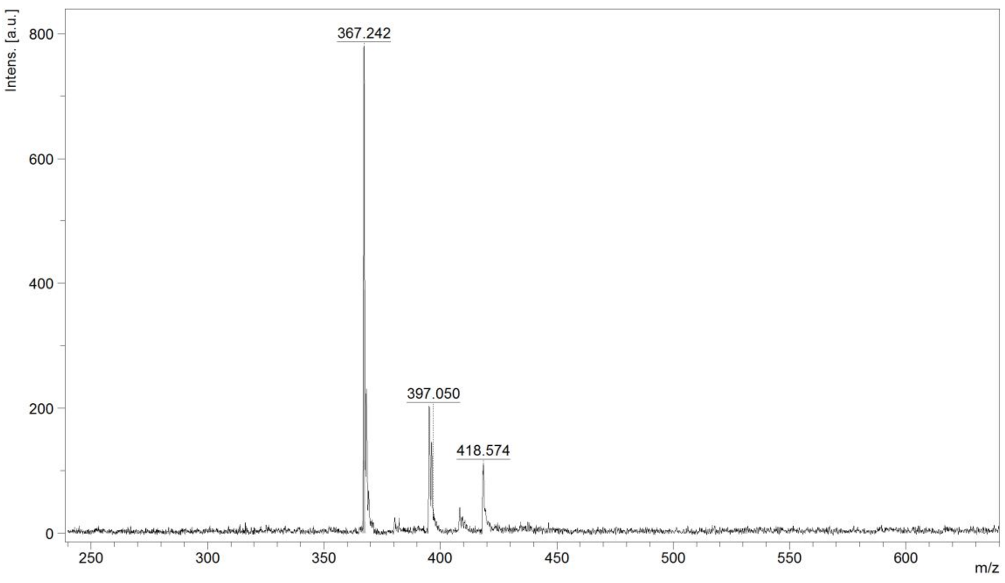

Figure S5. Mass spectrum of the compound **3**.

The mass spectrum of the compound **3** is provided in Figure S5. Upon analysis of the spectrum, the molecular ion peak $[M]^+$ at 397.050 m/z and the $[M+Na]^+$ peak at 418.574 m/z for compound 3, which has the molecular formula $C_{20}H_{19}O_6N_3$ and a calculated molecular weight of 397.38 g/mol, support the correctness of the expected structure of the compound **3**.

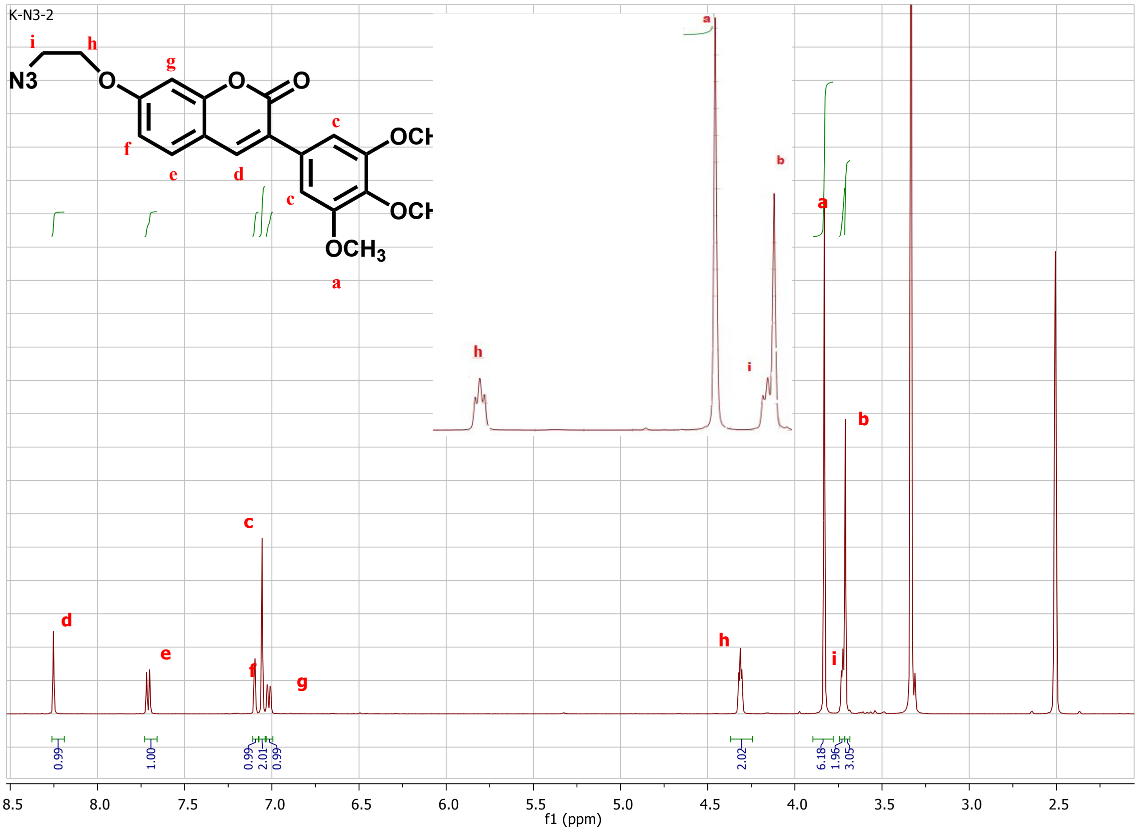

Figure S6. ^{1}H-NMR spectrum of the compound **3**.

In the ^{1}H-NMR spectrum of the compound **3** recorded in DMSO-d_6, the aromatic protons were observed in the range of 7.70-7.01 ppm. The He proton appeared as a doublet at 7.70 ppm. The Hf proton, which exhibited ortho coupling with the He proton, split into a doublet, and further split into a doublet of doublets due to meta coupling with the Hg proton, was observed at 7.05 ppm. The Hg proton appeared as a doublet at 7.05 ppm. The Hc protons were observed as a singlet at 7.01 ppm. The Ha and Hb protons of the methoxy groups attached to the meta and para positions of the phenyl ring at the 3-position of the coumarin structure were observed as singlets at 3.83 and 3.72 ppm, respectively. In the ^{1}H-NMR spectrum of the coumarin compound, the vinylic Hd proton was observed as a singlet at 8.25 ppm, downfield. The aliphatic CH_2 protons appeared as triplets at 3.83 and 3.72 ppm, splitting into each other. All

observed chemical shift values (δ), coupling constants (J), and integration values for this compound confirm the correctness of the structure of the compound **3**.

Characterizations of Compound 6 (Calix-Coumarin)

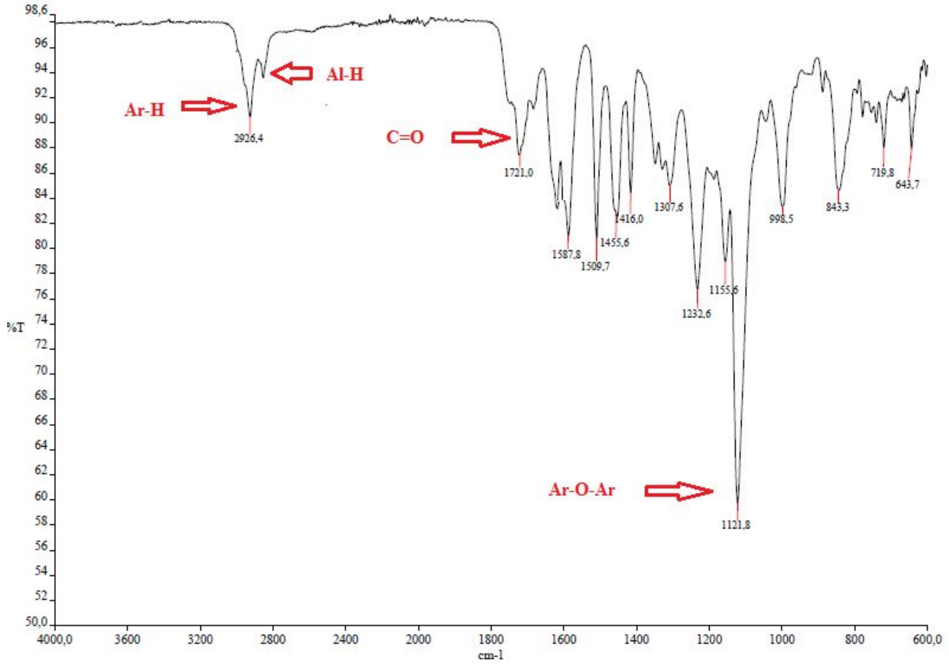

Figure S7. FT-IR Spectrum of **Calix-Coumarin**.

In the FT-IR spectrum of **Calix-Coumarin** derivative, an aromatic -CH peak was observed at 3010-2926 cm^{-1}, an aliphatic -CH peak was observed at around 2900 cm^{-1}, a carbonyl (C=O) peak was observed at 1721 cm^{-1}, and an ether (Ar-O-Ar) peak was observed at 1121.8 cm^{-1}. The absence of the azide peak at 2126 cm^{-1}, which is characteristic of compound **3**, as well as the absence of the peaks at 3285-3331 cm^{-1} (υ(C≡CH)) and 2117 cm^{-1} (υ(C≡C)), which are characteristic of compound **5**, further confirm the structure of the **Calix-Coumarin** derivative.

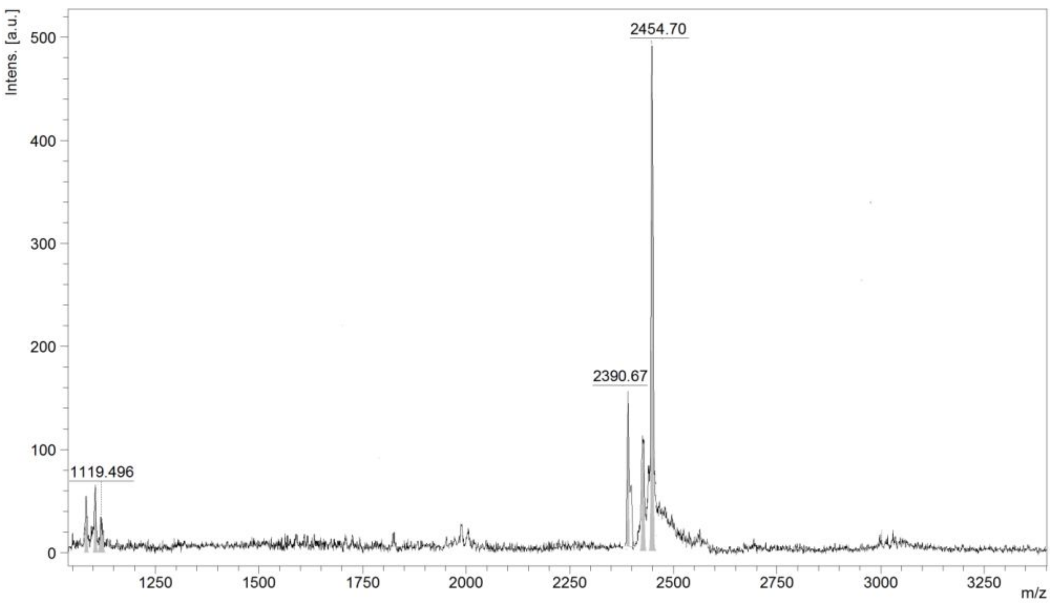

Figure S8. Mass spectrum of **Calix-Coumarin**.

The mass spectrum of **Calix-Coumarin** derivative is provided in Figure S8. Upon analysis of the spectrum, the molecular ion peaks $[M]^+$ at 2390.67 m/z and $[M+Cu]^+$ at 2454.70 m/z for **Calix-Coumarin** derivative, which has the molecular formula $C_{136}H_{140}N_{12}O_{28}$ and a calculated molecular weight of 2390.62 g/mol, support the correctness of the expected structure of the **Calix-Coumarin** derivative.

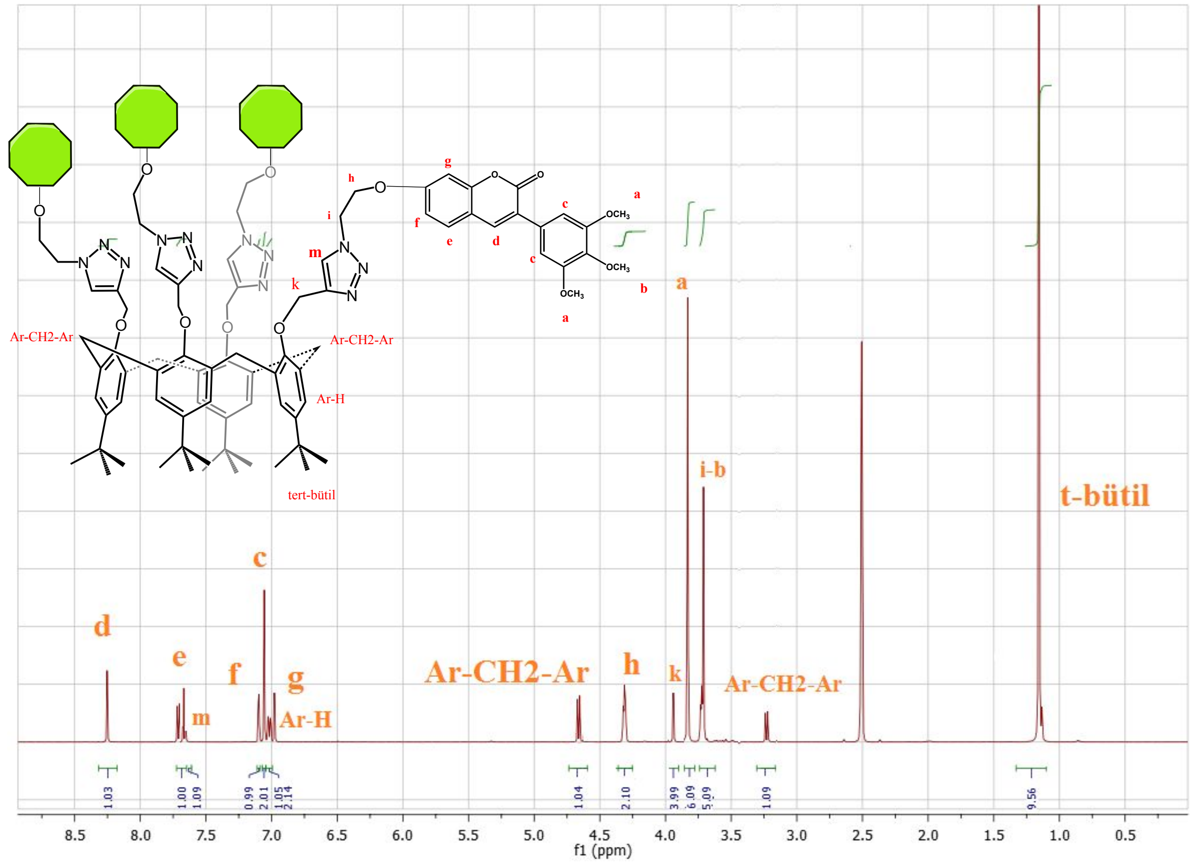

Figure S9. ^{1}H-NMR pectrum of the **Calix-Coumarin**.

The ^{1}H NMR spectrum of the calix–coumarin compound reflects its highly aromatic molecular architecture. The aromatic protons associated with the coumarin moiety and the triazole ring are observed as multiplet signals in the region of 8.27–7.04 ppm. The aromatic protons of the calixarene framework appear as characteristic signals centered around 7.00 ppm. The methylene bridge protons (Ar–CH_2–Ar) of the calixarene unit are clearly resolved as two distinct doublets at 4.62 and 3.15 ppm, which is indicative of a rigid cone conformation of the calixarene scaffold. Furthermore, the tert-butyl substituents attached to the calixarene rings give rise to sharp singlet signals at 1.16 ppm, as expected. Overall, the observed chemical shift values (δ), coupling constants (J), and integral ratios are fully consistent with the proposed molecular structure, thereby confirming the successful synthesis of the calix–coumarin sensor.
